# The safety of colorectal cancer surgery during the COVID-19: a systematic review and meta-analysis

**DOI:** 10.3389/fonc.2023.1163333

**Published:** 2023-07-17

**Authors:** Qiuxiang Wang, Ruike Wu, Juan Wang, Yilin Li, Qin Xiong, Fengjiao Xie, Peimin Feng

**Affiliations:** ^1^ Department of Gastroenterology, Hospital of Chengdu University of Traditional Chinese Medicine, Chengdu, Sichuan, China; ^2^ Department of Traditional Chinese Medicine, The Central Hospital of Guangyuan City, Sichuan, China

**Keywords:** colorectal cancer, surgery, COVID-19 pandemic, meta-analysis, safety

## Abstract

**Background:**

The ongoing coronavirus disease 2019 (COVID-19) pandemic has placed unprecedented pressure on the healthcare systems. This study evaluated the safety of colorectal cancer (CRC) surgery during the COVID-19 pandemic.

**Methods:**

A systematic review and meta-analysis were performed according to Preferred Reporting Items for Systematic Reviews and Meta-Analyses (PRISMA) guidelines (PROSPERO ID: CRD 42022327968). Relevant articles were systematically searched in the PubMed, Embase, Web of Science, and Cochrane databases. The postoperative complications, anastomotic leakage, postoperative mortality, 30-day readmission, tumor stage, total hospitalization, postoperative hospitalization, preoperative waiting, operation time, and hospitalization in the intensive care unit (ICU) were compared between the pre-pandemic and during the COVID-19 pandemic periods.

**Results:**

Among the identified 561 articles, 12 met the inclusion criteria. The data indicated that preoperative waiting time related to CRC surgery was higher during the COVID-19 pandemic (MD, 0.99; 95%CI, 0.71–1.28; p < 0.00001). A similar trend was observed for the total operative time (MD, 25.07; 95%CI, 11.14–39.00; p =0.0004), and on T4 tumor stage during the pandemic (OR, 1.77; 95%CI, 1.22–2.59; p=0.003). However, there was no difference in the postoperative complications, postoperative 90-day mortality, anastomotic leakage, and 30-day readmission times between pre-COVID-19 pandemic and during the COVID-19 pandemic periods. Furthermore, there was no difference in the total hospitalization time, postoperative hospitalization time, and hospitalization time in ICU related to CRC surgery before and during the COVID-19 pandemic.

**Conclusion:**

The COVID-19 pandemic did not affect the safety of CRC surgery. The operation of CRC during the COVID-19 pandemic did not increase postoperative complications, postoperative 90-day mortality, anastomotic leakage, 30-day readmission, the total hospitalization time, postoperative hospitalization time, and postoperative ICU hospitalization time. However, the operation of CRC during COVID-19 pandemic increased T4 of tumor stage during the COVID-19 pandemic. Additionally, the preoperative waiting and operation times were longer during the COVID-19 pandemic. This provides a reference for making CRC surgical strategy in the future.

**Systematic review registration:**

https://www.crd.york.ac.uk/prospero/, identifier CRD42022327968.

## 1 Introduction

Colorectal cancer (CRC) is the fourth most prevalent cancer globally and the second leading cause of cancer-related deaths, accounting for 6.1% of the world’s morbidity and 9.2% of cancer-related deaths (1). The incidence of CRC in developing countries is rising (2). Factors such as lifestyle changes, including dietary change, lesser engagement in physical activity, and an increase in sedentary behavior, have increased CRC incidence. It is projected that by 2035, there will be 2.5 million new CRC cases yearly (3). CRC is profoundly an asymptomatic disease. Thus, the cancer is usually diagnosed in the advanced stage (4). Colonoscopy is the first choice for colon cancer diagnosis. Histology remains as the standard method for the pathological staging of CRC, which informs the subsequent treatment approach (4). Surgery, neoadjuvant radiotherapy, and adjuvant chemotherapy are the common methods for CRC treatment (5). Surgery is the standard CRC treatment modality. However, the ongoing coronavirus disease 2019 (COVID-19) pandemic has drastically challenged the safety of CRC surgery.

Severe acute respiratory syndrome coronavirus-2 (SARS-CoV-2), the causative pathogen for COVID-19, first appeared in Wuhan, China, in December 2019. After the rapid spread of the virus, COVID-19 was declared a pandemic by the World Health Organization in March 2020 (6). The morbidity and mortality rates from the COVID-19 epidemic were very high. Given the high number of individuals affected by the virus, the health systems were compromised. The COVID-19 pandemic has created a public health crisis. As of 24 July 2022, there were more than 567 million confirmed cases and more than 6.3 million deaths from the COVID-19 pandemic worldwide. The COVID-19 pandemic has transcendently affected many healthcare systems worldwide. The pandemic has also delayed CRC surgery. Particularly, during the COVID-19 pandemic, an average of one in four (23.8% [268 of 1,128]) CRC patients died within 30 days, and approximately half (51.2% [577]) of them developed major pulmonary complications (7). Nevertheless, a recent review suggested that during the COVID-19 epidemic, the delay of elective surgery for CRC patients should not exceed 4 weeks because delayed surgery treatment is linked to poor prognosis (8).

However, there is no consensus concerning the safety of CRC surgery during the COVID-19 pandemic. Several studies have demonstrated that the pandemic is ineffective during CRC surgical procedures (9–12). However, a related study showed that mortality rates related to CRC elective surgery slightly increased during the COVID-19 pandemic (from 0.9% to 1.2%, p = 0.06). The mortality rates due to emergency surgery also significantly increased (from 5.6% to 8.9%, p = 0.003) (13). COVIDSurg Collaborative report revealed that mortality rates were lower in CRC patients who underwent elective surgery without an anastomotic leak or SARS-CoV-2 infection (14/1601, 0.9%) than in elective colorectal cancer surgery patients with both anastomotic leakage and SARS-CoV-2 (5/13, 38.5%) (14). In addition, a separate study demonstrated that the rate of postoperative complications in patients undergoing CRC surgery was higher in the COVID-19 pandemic group (15). Therefore, assessing the safety of colorectal cancer surgery during the COVID-19 pandemic is necessary. There are no meta-analyses reports so far on the safety of CRC surgery during the COVID-19 pandemic. Therefore, a meta-analysis of cohort studies was performed to evaluate the safety of CRC surgery during the COVID-19 pandemic. The purpose of this study is to compare the safety of colorectal cancer surgery before the COVID-19 pandemic and during the COVID-19 pandemic.

## 2 Materials and methods

### 2.1 Search strategy

This systematic review and meta-analysis were performed according to the Preferred Reporting Items for Systematic Reviews and Meta-Analyses (PRISMA) 2020 (16) and Assessing the Methodological Quality of Systematic Reviews (AMSTAR) guidelines, and the data were entered into PROSPERO under the registration number CRD42022327968. Relevant articles were systematically searched in PubMed, Embase, The Cochrane Library, and the Web of Science databases for relevant studies published between December 2019 and May 2022. The three sets of search terms used included “Colorectal cancer,” “COVID-19 pandemic,” and “Surgery.” The latest or most complete copy was used for articles updated multiple times. The detailed search strategies are shown in **Supplementary Appendix A**.

### 2.2 Study selection

**Figure 1:**
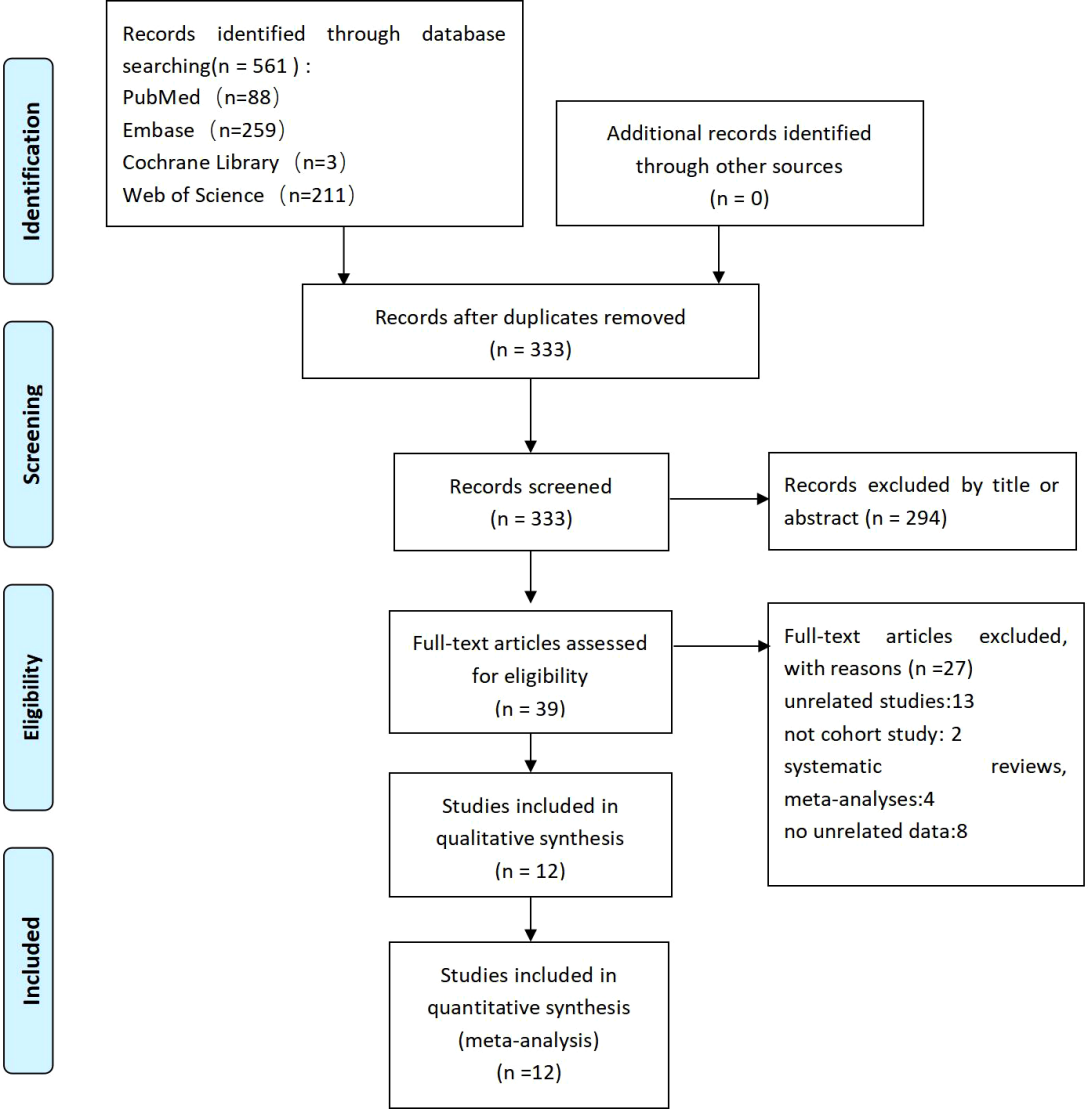
Flow diagram of the search method and selection process.

The selection of relevant studies was performed independently by two authors. The obtained articles were imported into EndNoteX9 for sorting. After removing duplicates, the title and abstract of the remaining articles were evaluated. Irrelevant literature were then removed, and afterward, the full text of the remaining studies were evaluated. Disagreements between the two authors were arbitrated by a third author. The research selection process is shown in **Figure 1**.

### 2.3 Inclusion and exclusion criteria

Studies that have met the following criteria were included: (1) cohort studies, (2) the study population comprised CRC patients, (3) compared CRC surgery safety before and during the COVID-19 pandemic, (4) on any CRC surgical type (minimally invasive or open surgery), and (5) the primary outcome indicators assessed included incidence of postoperative complications and/or postoperative anastomotic leakage, postoperative 90-day mortality, total hospitalization time, postoperative hospitalization time, preoperative waiting time, operation time, hospitalization time in ICU, and 30-day readmission time, and tumor stage. Studies were included if at least one of the perioperative outcomes was reported.

The following studies were excluded: (1) on CRC surgery during the COVID-19 pandemic but excluded COVID-19 patients; (2) duplicates; (3) meta-analyses, reviews, case reports, editorials, and letters; (4) with unsuitable data; (5) with Newcastle–Ottawa Scale (NOS) scores lower than 5 points; and (6) published in other languages other than English.

### 2.4 Data extraction

Two reviewers (FX and QX) independently extracted data from the included studies and inputted the extracted data into Excel sheets. Any differences are resolved through discussion until a consensus was reached. Further controversy was arbitrated by the corresponding author (PF). The following information was extracted: (1) detailed features included in the studies, namely, the first author’s name, year of publication, country, research design, research scale, age, and gender; (2) main results, namely, postoperative complications, postoperative anastomotic leakage, and postoperative mortality; and (3) secondary outcomes, namely, total hospital stay, postoperative stay, preoperative waiting time, operation time, hospitalization time in ICU and 30-day readmission, and tumor stage.

### 2.5 Quality assessment

Two authors (RW and JW) independently evaluated the quality of each included article on the basis of The Newcastle–Ottawa Scale (NOS) (17). Any disagreements were resolved through discussion. The quality of the articles included is evaluated from the following three evaluation categories: selection, comparability, and exposure/results (18). This scale has three parameters and eight items, with a total score of 9 points. A scores of ≤3 is usually considered as low quality, score of 4 or 5 is considered as medium quality, and score of ≥6 is usually considered as high quality (19).

### 2.6 Statistical analysis

We used the Review Manager software (version 5.4) for meta-analysis. Sensitivity analysis, funnel plot, and Egger’s test were performed with Stata software (version 16.0). If the included article reported outcomes in medians and interquartile ranges (IQR), the method described by Wan et al. was used to calculate the mean and standard deviation (SD) (20, 21). If the included article reported outcomes in medians, maximum, and minimum, the method described by Hozo et al. was used to calculate the mean and SD (22). The results were presented as odds ratio (OR) with 95% confidence intervals (CIs) for dichotomous data. Mean difference (MD) with 95% confidence intervals (CIs) were used for continuous data. A random-effects model was used in all meta-analyses (23, 24). p<0.05 was considered statistically significant. We assessed the heterogeneity by using the I^2^ test developed by Higgins (25). By omitting a single study and calculating the summary data of the remaining studies, sensitivity analysis is conducted to assess the impact of each included study on the summary data. Evidence of publication bias was evaluated by applying Egger test to funnel plots, in which ≥10 studies were available (26).

## 3 Results

### 3.1 Literature search

From these four electronic databases, we initially collected 561 research articles closely related to the above topics. After preliminary screening and review, 228 studies were excluded as duplicate records, and 294 studies were excluded from the title or abstract. Moreover, after carefully reading, reviewing, and confirming the full-text content, a total of 12 studies (10, 12, 13, 15, 27–34) were finally included to form this meta-analysis. A flow chart of article screening and selection processes is shown in **Figure 1**.

### 3.2 Characteristics of the studies included

The detailed characteristics of studies included in the meta-analysis are summarized in **Table 1**. Overall, 12 articles on 15,232 patients were included in this meta-analysis. Of the 15,232 patients, 4,025 underwent CRC surgery during the COVID-19 pandemic (26.4%), and 11,207 underwent CRC surgery before the COVID-19 pandemic (73.6%). Of the 12 studies included in this meta-analysis, two (28, 31) were performed in China, two (15, 27) in Turkey, two (13, 29) in the UK, two (32, 33) in Romania, and one each in Austria (30), Serbia (10), Italy (34), and Denmark (12). The perioperative outcomes of patients in the articles are summarized in **Table 2**.

**Table 1:** The characteristics of each included study.

Author	Year	Country	Study design	Cancer type	Study size	Age ± SD/(IQR)/ mean(min-max)/median (IQR)	Gender Male/Female
Xu et al. (31)	2021	China	Retrospective cohort	CRC	PCG:828 CG:710	PCG:NA CG:NA	PCG:518/310 CG:438/272
Uyan et al. (15)	2022	Turkey	Retrospective cohort	CRC	PCG:56 CG:48	PCG:64.9 (41-89) CG:63.2 (22-90)	PCG:32/24 CG:31/17
Cui et al. (28)	2022	China	Retrospective cohort	CRC	PCG: 104(2018) 101(2019) CG:67	PCG: 64.3 ± 11.2(2018) 67.0 ± 12.0(2019) CG:67.1 ± 11.4	PCG: 54/50(2018) 57/44(2019) CG:44/23
Kuryba et al. (13)	2021	U K	Retrospective cohort	CRC	PCG: Elective surgery: 8,774; Emergency surgery: 1,493 CG: Elective surgery: 2,267 Emergency surgery: 526	PCG:NA CG:NA	PCG:NR CG:NR
Tschann et al. (30)	2021	Austria	Retrospective cohort	CRC	PCG: 1st half of 2019: 46 2nd half of 2019:25 CG: 1st half of 2020:29 2nd half of 2020:34	PCG: 1st half of 2019:67.5 ± 11.7 2nd half of 2019:64.0 ± 15.7 CG: 1st half of 2020:69.5 ± 13.1 2nd half of 2020:68.1 ± 13.2	PCG: 1st half of 2019:27/19 2nd half of 2019:15/10 CG: 1st half of 2020:12/17 2nd half of 2020:22/12
Rashid et al. (29)	2021	U K	Retrospective cohort	CRC	PCG:10 CG:22	PCG:69 ± 13 CG:74 ± 7	PCG:7/3 CG:16/6
Ferahman et al. (27)	2020	Turkey	Retrospective cohort	CRC	PCG:27 CG:35	PCG:65.3 ± 13.48 CG:61.3 ± 10.86	PCG:17/10 CG:22/13
Kiss et al. (32)	2022	Romania	Retrospective cohort	CRC	PCG:160 CG:142	PCG:67.66 ± 12.25 CG:67.08 ± 10.9	PCG:111/49 CG:87/55
Smith et al. (12)	2021	Denmark	Retrospective cohort	CRC	PCG: CC:872 RC:304 CG: CC:509 RC:172	PCG: CC : NA RC : NA CG: CC : NA RC : NA	PCG: CC : NA RC : NA CG: CC : NA RC : NA
Radulovic et al. (10)	2021	Serbia	Retrospective cohort	CRC	PCG:152 CG:49	PCG:67.11 ± 11.621 CG: 67.41 ± 10.378	PCG:87/65 CG: 22/27
Feier et al. (33)	2022	Romania	Retrospective cohort	CRC	PCG: 2016–2017:40 2018-2019:49 CG:29	PCG:NA CG:NA	PCG:NA CG:NA
Losurdo et al. (34)	2022	Italy	Retrospective cohort	CRC	PCG:132 CG:118	PCG:77.0 (47–94) CG: 77.5 (32–93)	PCG:68/64 CG: 57/61

NA, not applicable, NR, not reported; min, minimum; max, maximum; CRC, colorectal cancer; PCG, pre-COVID-19 group, CG, COVID-19 group.

**Table 2:** The outcomes of each included study.

Author	Postoperative complications	Anastomotic leakage	Mortality	30-day readmission	The total hospital stay mean ± SD	Postoperative ICU stay mean ± SD	Postoperative hospital stay mean ± SD	Preoperative waiting time mean ± SD	Operation time mean ± SD
Xu et al. (31)	PCG:42 CG:28	PCG : NR CG : NR	PCG : NR CG : NR	PCG : NR CG : NR	PCG:11 ± 4 CG:13.2 ± 4.5	PCG : NR CG : NR	PCG:7.2 ± 2.8 CG:8.4 ± 3.1	PCG:3.8 ± 2.8 CG:4.8 ± 3	PCG : NR CG : NR
Uyan et al. (15)	PCG:11 CG:20	PCG:1 CG:1	PCG:3 CG:4	PCG : NR CG : NR	PCG:9.3 ± 13.25 CG:10.8 ± 13.75	PCG : NR CG : NR	PCG : NR CG : NR	PCG : NR CG : NR	PCG : NR CG : NR
Cui et al. (28)	PCG: 13(2018) 8(2019) CG:9	PCG: NR(2018) NR(2019) CG : NR	PCG: 0(2018) 0(2019) CG:0	PCG: NR(2018) NR(2019) CG : NR	PCG: NR(2018) NR(2019) CG : NR	PCG: NR(2018) NR(2019) CG : NR	PCG: 12.1 ± 9.1(2018) 9.2 ± 4.2(2019) CG:9.6 ± 3.7	PCG: 9.2 ± 6.3(2018) 8.1 ± 4.3(2019) CG:8.9 ± 4.9	PCG: 226.3 ± 80.3(2018) 206.21 ± 63.64(2019) CG:245.22 ± 88.94
Kuryba et al. (13)	PCG : NR CG : NR	PCG : NR CG : NR	PCG : NR CG : NR	PCG: Elective surgery: 895 Emergency surgery: 163 CG: Elective surgery: 240 Emergency surgery: 62	PCG : NR CG : NR	PCG : NR CG : NR	PCG : NR CG : NR	PCG : NR CG : NR	PCG : NR CG : NR
Tschann et al. (30)	PCG: 1st half of 2019:9 2nd half of 2019:5 CG: 1st half of 2020:3 2nd half of 2020:8	PCG: 1st half of 2019:6 2nd half of 2019:4 CG: 1st half of 2020:0 2nd half of 2020:3	PCG: 1st half of 2019:NR 2nd half of 2019:NR CG: 1st half of 2020:NR 2nd half of 2020:NR	PCG: 1st half of 2019:NR 2nd half of 2019:NR CG: 1st half of 2020:NR 2nd half of 2020:NR	PCG: 1st half of 2019: 13.6 ± 9.1 2nd half of 2019: 14.9 ± 17.2 CG: 1st half of 2020: 12.2 ± 7.8 2nd half of 2020: 15 ± 13.5	PCG: 1st half of 2019:NR 2nd half of 2019:NR CG: 1st half of 2020:NR 2nd half of 2020:NR	PCG: 1st half of 2019:NR 2nd half of 2019:NR CG: 1st half of 2020:NR 2nd half of 2020:NR	PCG: 1st half of 2019:NR 2nd half of 2019:NR CG: 1st half of 2020:NR 2nd half of 2020:NR	PCG: 1st half of 2019:NR 2nd half of 2019:NR CG: 1st half of 2020:NR 2nd half of 2020:NR
Rashid et al. (29)	PCG:10 CG:22	PCG:0 CG:0	PCG:0 CG:0	PCG:0 CG:0	PCG:NR CG:NR	PCG:1 ± 3 CG:0.1 ± 0.5	PCG:8 ± 9 CG:5 ± 2	PCG:NR CG:NR	PCG:NR CG:NR
Ferahman et al. (27)	PCG:4 CG:5	PCG:1 CG:1	PCG:2 CG:9	PCG:NR CG:NR	PCG:9 ± 8.22 CG:7.8 ± 6.01	PCG:NR CG:NR	PCG:NR CG:NR	PCG:NR CG:NR	PCG:163.4 ± 55.42 CG:173.4 ± 46.55
Kiss et al. (32)	PCG:NR CG:NR	PCG:NR CG:NR	PCG:12 CG:16	PCG:NR CG:NR	PCG:10.3 ± 5.066 CG:11 ± 7.659	PCG:2.962 ± 2.676 CG:3.792 ± 3.922	PCG:NR CG:NR	PCG:NR CG:NR	PCG : NR CG : NR
Smith et al. (12)	PCG: CC:119 RC:71 CG: CC:65 RC:31	PCG: CC:24 RC:14 CG: CC:13 RC:5	PCG: CC:27 RC:5 CG: CC:14 RC:5	PCG: CC : NR RC : NR CG: CC : NR RC : NR	PCG: CC : NR RC : NR CG: CC : NR RC : NR	PCG: CC : NR RC : NR CG: CC : NR RC : NR	PCG: CC : NR RC : NR CG: CC : NR RC : NR	PCG: CC : NR RC : NR CG: CC : NR RC : NR	PCG: CC : NR RC : NR CG: CC : NR RC : NR
Radulovic et al. (10)	PCG:NR CG:NR	PCG:NR CG:NR	PCG:NR CG:NR	PCG:9 CG:4	PCG:NR CG:NR	PCG:NR CG:NR	PCG:10.77 ± 6.09 CG:9.58 ± 3.64	PCG:NR CG:NR	PCG:NR CG:NR
Feier et al. (33)	PCG:NR CG:NR	PCG:NR CG:NR	PCG: 9(2016–2017) 9(2018–2019) CG:10	PCG:NR CG:NR	PCG:NR CG:NR	PCG:NR CG:NR	PCG:NR CG:NR	PCG:NR CG:NR	PCG:NR CG:NR
Losurdo et al. (34)	PCG:NR CG:NR	PCG:NR CG:NR	PCG:NR CG:NR	PCG:NR CG:NR	PCG:NR CG:NR	PCG:NR CG:NR	PCG:NR CG:NR	PCG:NR CG:NR	PCG:180 ± 68.33 CG: 205 ± 63.33

NA, not applicable; NR, not reported; PCG, pre-COVID-19 group; CG, COVID-19 group; CC, colon cancer; RC, rectal cancer.

### 3.4 Quality of the included studies

The quality of the 12 retrospective cohort studies was assessed based on NOS. Among them, one article (34) had five stars, one article (32) had six stars, five articles (15, 27, 30, 31, 33) had seven stars, three articles (10, 12, 28)had eight stars, and two studies (13, 29)had nine stars. Thus, all but one of the articles (34) were of high quality. Detailed quality assessment results are shown in **Table 3**.

**Table 3:** Quality assessment of included studies.

Study ID	Selection (out of 4)				Comparability (out of 2)	Outcomes (out of 3)			Total
	①	②	③	④		⑤	⑥	⑦	
Xu et al., 2021 (31)	*	*	*	*	**	*	**–**	**–**	**7**
Uyan et al., 2022 (15)	*	*	*	*	**	*	**–**	**–**	7
Cui et al., 2021 (28)	*	*	*	*	*	*	*	*	8
Kuryba et al., 2021 (13)	*	*	*	*	**	*	*	*	9
Tschann et al., 2021 (30)	*	*	*	*	**	*	**–**	**–**	7
Rashid et al., 2021 (29)	*	*	*	*	**	*	*	*	9
Ferahman et al., 2020 (27)	*	*	*	*	**	*	**–**	**–**	7
Kiss et al., 2022 (32)	*	*	*	*	*	*	**–**	**–**	6
Smith et al., 2021 (12)	*	*	*	*	*	*	*	*	8
Radulovic et al. (10)	*	*	*	*	*	*	*	**–**	8
Feier et al. (33)	*	*	*	*	*	**–**	*	*	7
Losurdo et al. (34)	*	*	*	*	*	**–**	**–**	**–**	5

① Representativeness of exposed cohort; ② selection of non-exposed cohort; ③ ascertainment of exposure; ④ outcome not present at the start of the study; ⑤ assessment of outcomes; ⑥ length of follow-up; ⑦ adequacy of follow-up.

The symbol * and ** indicate the use of the Newcastle-Ottawa scale to evaluate the quality of included studies.

### 3.5 Primary surgical outcomes

#### 3.5.1 Postoperative complications

**Figure 2:**
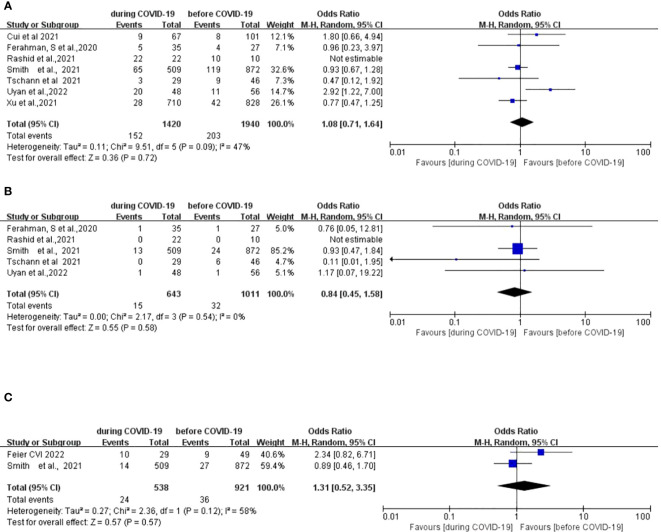
Forest plot of primary surgical outcomes. **(A)** Forest plot of postoperative complications before and during the COVID-19 pandemic. **(B)** Forest plot of postoperative anastomotic leakage before and during the COVID-19 pandemic. **(C)** Forest plot of postoperative 90-day mortality before and during the COVID-19 pandemic.

Seven studies (12, 15, 27–31) compared the postoperative complications between 1,940 patients who underwent surgery before the COVID-19,pandemic and 1,420 patients who underwent surgery during the COVID-19 pandemic. There was no significant difference in postoperative complications between the two groups (OR: 1.08; 95% CI: 0.74–1.64; p =0.72) (**Figure 2A**).

#### 3.5.2 Postoperative anastomotic leakage

Five studies (12, 15, 27, 29, 30) compared incidences of postoperative anastomotic leakage between 1,011 patients who underwent surgery before the COVID-19 pandemic and 643 patients who underwent surgery during the COVID-19 pandemic. There was no significant difference in the postoperative anastomotic leakage between the two groups (OR: 0.84; 95% CI: 0.45–1.58; p =0.58) (**Figure 2B**).

#### 3.5.3 Postoperative 90-day mortality

Two studies (12, 33) compared postoperative mortality rates between 921 patients who underwent surgery before the COVID-19 pandemic and 538 patients who underwent surgery during the COVID-19 pandemic. There was no significant difference in the postoperative 90-day mortality between the two groups (OR, 1.31; 95%CI, 0.52–3.35; p =0.57) (**Figure 2C**).

### 3.6 Secondary surgical outcomes

#### 3.6.1 Total hospitalization time

**Figure 3:**
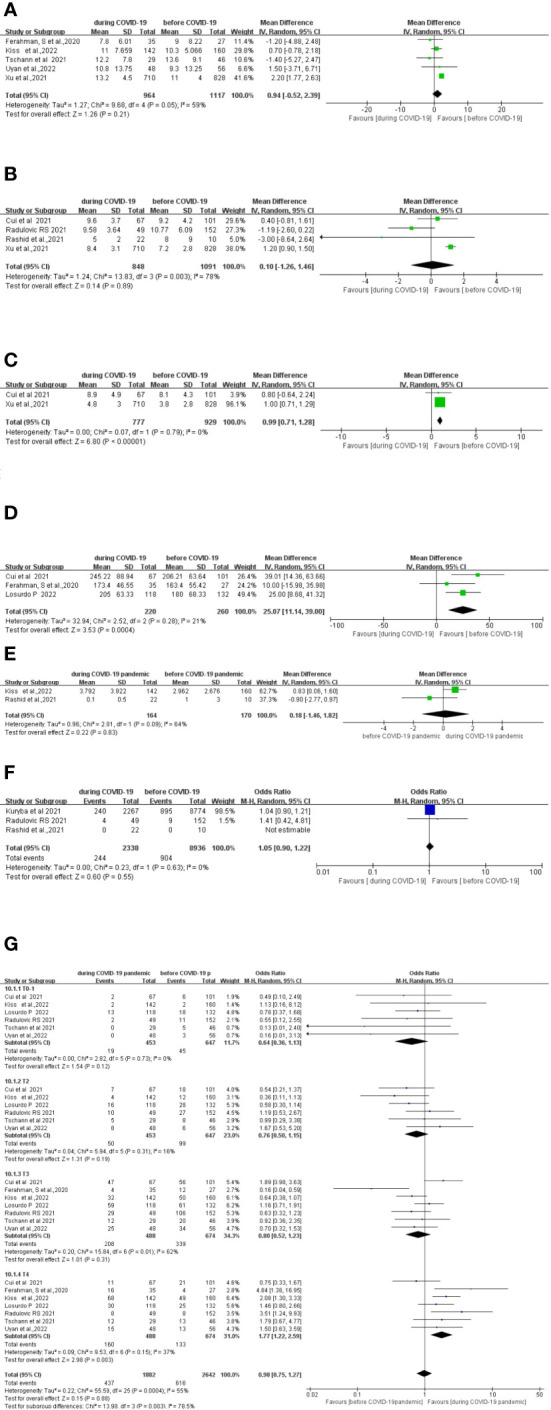
Forest plot of secondary surgical outcomes. **(A)** Forest plot of total hospital stay before and during the COVID-19 pandemic. **(B)** Forest plot of postoperative stay before and during the COVID-19 pandemic. **(C)** Forest plot of preoperative waiting time before and during the COVID-19 pandemic. **(D)** Forest plot of total operative time before and during the COVID-19 pandemic. **(E)** Forest plot of postoperative intensive care unit (ICU) stay before and during the COVID-19 pandemic. **(F)** Forest plot of postoperative 30-day readmission before and during the COVID-19 pandemic. **(G)** Forest plot of tumor stage before and during the COVID-19 pandemic.

Five studies (15, 27, 30–32)compared the length of hospital stay between 1,117 patients who underwent surgery before the COVID-19 pandemic and 964 patients who underwent surgery during the COVID-19 pandemic. No significant difference in pooled data was found between the two groups (MD, 0.94; 95% CI, −0.52–2.39; p =0.94) (**Figure 3A**).

#### 3.6.2 Postoperative hospitalization time

Four studies (10, 28, 29, 31) compared the length of the postoperative stay between 1,091 patients who underwent surgery before the COVID-19 pandemic with 848 patients who underwent surgery during the COVID-19 pandemic. There was no significant difference in the length of the postoperative stay between the two groups (MD, 0.10; 95%CI, −1.26–1.46; p =0.89) (**Figure 3B**).

#### 3.6.3 Preoperative waiting time

Two studies (28, 31) compared the difference in preoperative waiting time between 929 patients who underwent surgery before the COVID-19 pandemic and 777 patients who underwent surgery during the COVID-19 pandemic. Preoperative waiting time was significantly longer during the COVID-19 pandemic period (MD, 0.99; 95%CI, 0.71–1.28; p < 0.00001) (**Figure 3C**).

#### 3.6.4 Total operative time

Three studies (27, 28, 34) compared the total operative time between 260 patients who underwent surgery before the COVID-19 pandemic and 220 patients who underwent surgery during the COVID-19 pandemic. The total operative time was significantly longer during the COVID-19 pandemic (MD, 25.07; 95%CI, 11.14–39.00; p =0.0004) (**Figure 3D**).

#### 3.6.5 Postoperative intensive care unit stay

Two studies (29, 32) compared the length of the postoperative ICU stay between 170 patients who underwent surgery before the COVID-19 pandemic and 164 patients who underwent surgery during the COVID-19 pandemic. No significant difference in the length of the postoperative ICU stay between the two groups was found (MD, −0.18; 95%CI, −1.46–1.82; p =0.83) (**Figure 3E**).

#### 3.6.6 Postoperative 30-day readmission

Three studies (10, 13, 29) compared postoperative 30-day readmission between 8,936 patients who underwent surgery before the COVID-19 pandemic and 2,338 patients who underwent surgery during the COVID-19 pandemic. No significant difference in the postoperative 30-day readmission between the two groups was found (OR, 1.05; 95%CI, 0.90–1.22; p =0.55) (**Figure 3F**).

#### 3.6.7 Tumor stage

Seven studies (10, 15, 27, 28, 30, 32, 34) compared tumor stages between 488 patients who underwent surgery before the COVID-19 pandemic and 674 patients who underwent surgery during the COVID-19 pandemic. The T4 cases were significantly higher during COVID-19 period (OR, 1.77; 95%CI, 1.22–2.59; p=0.003). There was no significant difference in the T1, T2, and T3 cases between the pre-COVID-19 and during the COVID-19 pandemic periods (**Figure 3G**).

### 3.7 Publication bias

**Figure 4:**
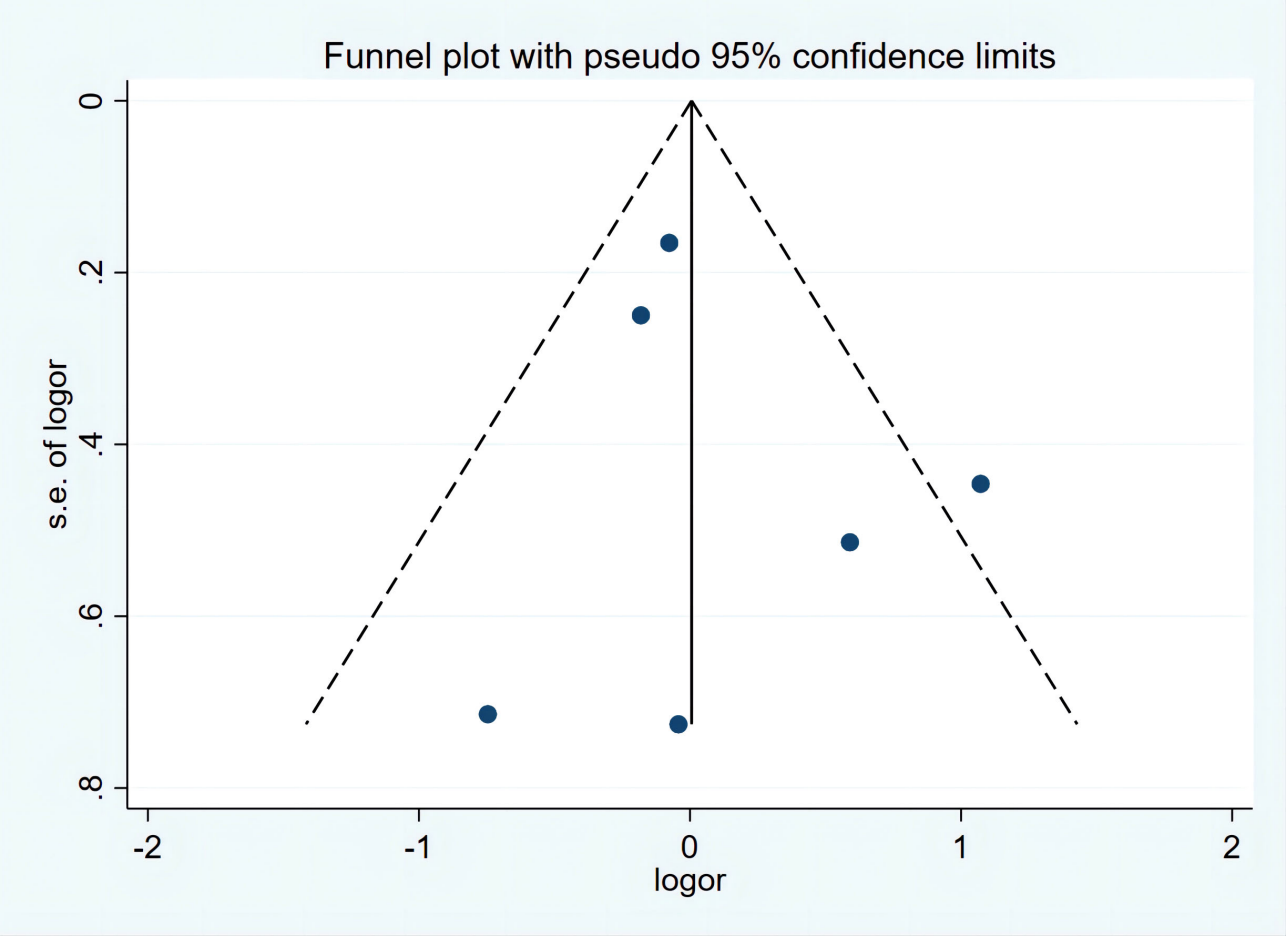
Funnel plot of postoperative complications.

The funnel plot of the postoperative complications revealed a slightly asymmetrical distribution (**Figure 4**). Nevertheless, Egger’s test indicated that there is no significant publication bias (p=0.549). Other surgical outcomes were not analyzed for publication bias because of insufficient data.

### 3.8 Sensitivity analysis

By removing the studies one by one, none of the studies changed the pooled data of the postoperative complications. The results of the sensitivity analysis are displayed in **Supplementary Appendix B**. Therefore, the results were relatively reliable and stable in meta-analysis.

## 4 Discussion

The study results showed that compared to the prior COVID-19 pandemic period, performing CRC surgery during the COVID-19 pandemic did not increase postoperative complications, anastomotic leakage, postoperative 90-day mortality, 30-day readmission, the total hospital stay, postoperative hospital stay, and postoperative ICU stay. However, CRC-related preoperative waiting and operation time were higher and longer, respectively, during the COVID-19 pandemic period. Besides, the operation of CRC during COVID-19 pandemic increased T4 of tumor stage during the COVID-19 pandemic. This provides a reference for us to formulate CRC surgical strategies in the future and enlightens us that in order to improve the long-term outcome of CRC, multiple stakeholders need to consider new strategies and invest appropriate resources to increase CRC cancer screening in line with the guidelines.

Postoperative mortality is one of the important indicators for the quality and safety of surgery and anesthesia. In their retrospective analysis in Turkey, Uyan et al. reported that the mortality rates in the pre-pandemic and the pandemic period were 5% and 8%, respectively, which were not significantly different (p = 0.209), despite higher mortality in the pandemic period than in the pre-pandemic period (15). Similarly, Kiss et al. reported that the mortality rates in the pre-pandemic and the pandemic cohorts were 7.5% and 11.3%, respectively, which were significantly different (32). In addition, in a national population-based study in England, Kuryba et al. showed that CRC emery surgery-related mortality increased markedly from 5.6% in the pre-pandemic period to 8.9% in the pandemic period (p = 0.003) (13). A recent study indicated that the 90-day postoperative mortality rate of colon cancer increased to 34.5% during the pandemic (33). However, results of the present meta-analysis demonstrated that the odds of postoperative 90-day mortality did not increase (p=0.21) during the pandemic from 4.0% in the pre-COVID-19 pandemic to 5.1% in the COVID-19 pandemic period. The data showed that CRC surgery was safe during the COVID-19 pandemic.

Further meta-analysis revealed that the preoperative waiting time was longer during the COVID-19 pandemic. Particularly, the preoperative waiting was 0.89 days longer during the pandemic than in the pre-pandemic period. An international, prospective cohort study of 20,006 adults (≥18 years) with 15 cancer types in 466 hospitals and 61 countries revealed that one in seven patients who were in regions with full lockdowns during the COVID-19 pandemic had significant preoperative delays (35). These findings highlight the adjustments for CRC diagnosis and treatment made during the COVID-19 pandemic. Xu et al. reported that preoperative waiting was significantly longer during the pandemic because a patient had to undergo thorough screening for coronavirus infection before admission (31). Preoperative delays could also be attributed to an increase in the neoadjuvant therapy utilization. A recent meta-analysis comparing the oncological outcomes between direct surgery and neoadjuvant therapy before surgery for T4 colon cancer revealed that pretreatment with neoadjuvant therapy improved margin-negative resection rates and increased the overall survival of the patients (36). A population-based study in England reported a 44% increase in neoadjuvant therapy uptake/prescription rate for rectal cancer during the pandemic era, and the long-course regimens were more preferred over short-course modalities (37).

Finally, we found that the operation time of CRC was longer during the COVID-19 period. Specifically, the operation time during the pandemic was 24.05 min longer than in the pre-pandemic period. The COVID-19 epidemic affected cancer screening. A recent systematic review and meta-analysis on the association between the COVID-19 pandemic and cancer screening showed that colorectal cancer screening reduced by 44.9% (95%CI, −53.8% to −36.1%) after May 2020 (was 23.4% lower between June and October [95% CI, −44.4% to −2.4%]) compared with before (38). Diagnosis and treatment delays allow the tumor to worsen (39, 40), complicating the corresponding surgery. Surgeries had to be rescheduled during the COVID-19 pandemic, prioritizing urgent procedures and non-deferrable oncological cases (41). The operation time is expected to increase because the cancer would be advanced, requiring complex surgery. During the COVID-19 pandemic, preliminary guidelines recommended against laparoscopic surgery to avoid putative risks of SARS-CoV2 transmission through aerosolization of the pneumoperitoneum. However, this recommendation was lifted as more knowledge on the coronavirus came to light (42). A decline in laparoscopic surgery and an increase in open surgery are one of the reasons for the longer operation time.

Postoperative complications, anastomotic leakage, 30-day readmission, the total hospital stay, postoperative hospital stay, and postoperative ICU stay time did not differ significantly between the pre-pandemic period and the pandemic period. On the one hand, the proportion of major complication during the pandemic was not significantly different from that of the control group from four studies (12, 29–31). Anastomotic leakage remains a frequent and severe complication after CRC surgery (43). Three studies showed that the incidence of anastomotic leakage was not significantly higher during the pandemic period (12, 27, 29). On the other hand, 30-day readmission, total hospital stay, postoperative hospital stay, and postoperative ICU stay time were affected by postoperative complications and anastomotic leakage. That explains why the 30-day readmission, total hospital stay, postoperative hospital stay, and postoperative ICU stay time were not significantly different between the pre-pandemic and during the pandemic period. This could be related to patient psychology, in which patients are apprehensive of a longer stay in the hospital for fear of coronavirus infection.

To our knowledge, this is the first meta-analysis on the safety of CRC surgery during the COVID-19 pandemic. This meta-analysis had some limitations. First, because most of the included studies were retrospective single-center cohort studies, selection and sampling biases cannot be ruled out. Second, we only studied tumor stage (T) of surgical pathology outcomes, but other pathological outcomes, such as nodal stages, and lymph node yield, were not. Third, only two to three studies provided data on preoperative waiting time, postoperative ICU stay, operation time, postoperative 90-day mortality, and 30-day readmission rate. Further studies on these aspects are needed to provide more solid evidence. In addition, the heterogeneity of the hospital stay, postoperative stay, and ICU stay was high. This may be related to the small number of articles included in the analysis. Lastly, only 12 articles on 15,232 patients were included in this meta-analysis. Thus, findings should be interpreted with caution.

## 5 Conclusions

The operation for CRC during the COVID-19 pandemic was safe. Performing CRC surgery during the COVID-19 pandemic did not increase the rate of postoperative complications, anastomotic leakage, postoperative 90-day mortality, 30-day readmission, total hospital stay, postoperative hospital stay, and postoperative ICU stay. However, the preoperative waiting and operation time was longer. In addition, there are more patients in T4 tumor stage during the COVID-19 pandemic. This provides a reference for making CRC surgical strategy in the future.

## Data availability statement

The original contributions presented in the study are included in the article/**Supplementary Material**. Further inquiries can be directed to the corresponding author.

## Author contributions

QW conceived and designed this meta-analysis scheme and completed the drafts of the manuscript. YL designed the search strategies. FX and RW selected the eligible literatures. QX and FX independently extracted data. RW and JW independently evaluated the risk of bias. QW conducted data analysis. PF arbitrated any agreements in the process of meta-analysis. All authors contributed to the article and approved the submitted version.
