# Supplementary material for: The safety of colorectal cancer surgery during the COVID-19: a systematic review and meta-analysis
Source: Front Oncol. 2023 Jul 17;13:1163333. doi: 10.3389/fonc.2023.1163333 (PMC10390253; doi:10.3389/fonc.2023.1163333)
Supplement: Supplementary file 1 [file DataSheet_1.docx]

Appendix A – full search strategy

PubMed：

Search: **((((("COVID-19"[Mesh]) OR (COVID-19 pandemic[Title/Abstract])) OR ((COVID 19 pandemic[Title/Abstract]) OR (pandemic, COVID-19[Title/Abstract]))) OR (COVID-19 pandemics[Title/Abstract])) AND (("Colorectal Neoplasms"[Mesh]) OR (((((((((((((((Colorectal Neoplasm[Title/Abstract]) OR ('Neoplasm, Colorectal'[Title/Abstract])) OR ('Neoplasms, Colorectal'[Title/Abstract])) OR (Colorectal Tumors[Title/Abstract])) OR (Colorectal Tumor[Title/Abstract])) OR ('Tumor, Colorectal'[Title/Abstract])) OR ('Tumors, Colorectal'[Title/Abstract])) OR (Colorectal Cancer[Title/Abstract])) OR ('Cancer, Colorectal'[Title/Abstract])) OR ('Cancers, Colorectal'[Title/Abstract])) OR (Colorectal Cancers[Title/Abstract])) OR (Colorectal Carcinoma[Title/Abstract])) OR ('Carcinoma, Colorectal'[Title/Abstract])) OR ('Carcinomas, Colorectal'[Title/Abstract])) OR (Colorectal Carcinomas[Title/Abstract]))))) AND (("surgery" [Subheading]) OR (((((((operative therapy[Title/Abstract]) OR (operative procedures[Title/Abstract])) OR (operations[Title/Abstract])) OR (perioperative procedures[Title/Abstract])) OR (intraoperative procedures[Title/Abstract])) OR (peroperative procedures[Title/Abstract])) OR (preoperative procedures[Title/Abstract])))**

Embase:

#1 'covid 19 pandemics':ti,ab,kw OR 'covid-19 pandemic':ti,ab,kw OR 'covid 19pandemic':ti,ab,kw OR 'covid-19 pandemics':ti,ab,kw

#2 'colorectal tumor'/exp

#3 'colorectal neoplasm':ti,ab,kw OR 'neoplasm, colorectal':ti,ab,kw OR 'neoplasms, colorectal':ti,ab,kw OR 'colorectal tumors':ti,ab,kw OR 'colorectal tumor':ti,ab,kw OR 'tumor, colorectal':ti,ab,kw OR 'tumors, colorectal':ti,ab,kw OR 'colorectal cancer':ti,ab,kw OR 'cancer, colorectal':ti,ab,kw OR 'cancers, colorectal':ti,ab,kw OR 'colorectal cancers':ti,ab,kw OR 'colorectal carcinoma':ti,ab,kw OR 'carcinoma, colorectal':ti,ab,kw OR 'carcinomas, colorectal':ti,ab,kw OR 'colorectal carcinomas':ti,ab,kw

#4 #2OR#3

#5 'surgery'/exp

#6 'surgery':ti,ab,kw OR 'operative therapy':ti,ab,kw OR 'operative procedures':ti,ab,kw OR 'operations':ti,ab,kw OR 'perioperative procedures':ti,ab,kw OR 'intraoperative procedures':ti,ab,kw OR 'peroperative procedures':ti,ab,kw OR 'preoperative procedures':ti,ab,kw

#7 #5OR#6

#8 #1 AND #4 AND #7

Web of Science:

#1 ((((TS=(COVID-19 pandemics)) OR TS=(COVID-19)) OR TS=(COVID-19 pandemic)) OR TS=(pandemic, COVID-19)) OR TS=(COVID 19 pandemic)

#2 (((((((((((((((TS=(Colorectal Neoplasms)) OR TS=(Colorectal Neoplasm)) OR TS=(Neoplasm, Colorectal)) OR TS=(Neoplasms, Colorectal)) OR TS=(Colorectal Tumors)) OR TS=(Colorectal Tumor)) OR TS=(Tumor, Colorectal)) OR TS=(Tumors, Colorectal)) OR TS=(Colorectal Cancer)) OR TS=(Cancer, Colorectal)) OR TS=(Cancers, Colorectal)) OR TS=(Colorectal Cancers)) OR TS=(Colorectal Carcinoma)) OR TS=(Carcinoma, Colorectal)) OR TS=(Carcinomas, Colorectal)) OR TS=(Colorectal Carcinomas)

#3 (((((((TS=(surgery)) OR TS=(operative therapy)) OR TS=(operative procedures)) OR TS=(operations)) OR TS=(perioperative procedures)) OR TS=(intraoperative procedures)) OR TS=(peroperative procedures)) OR TS=(preoperative procedures)

#4 #1AND#2AND#3

Cochrane central register of trials:

 #1 colorectal neoplasms: ME

#2 (colorectal): ti,ab,kw and (cancer or neoplas* or carcinoma*)) : ti,ab,kw

#3 #1or#2

#4 (covid19pandemics or covid-19pandemic or covid 19 pandemic or covid-19 pandemics):ti,ab,kw

#5 (surgery* or operative therapy or operations*):ti,ab,kw

#6 #3 and #4 and #5
