# Supplementary figures and images for: The safety of colorectal cancer surgery during the COVID-19: a systematic review and meta-analysis
Source: Front Oncol. 2023 Jul 17;13:1163333. doi: 10.3389/fonc.2023.1163333 (PMC10390253; doi:10.3389/fonc.2023.1163333)

Supplementary: Appendix B: sensitivity analysis of postoperative complications


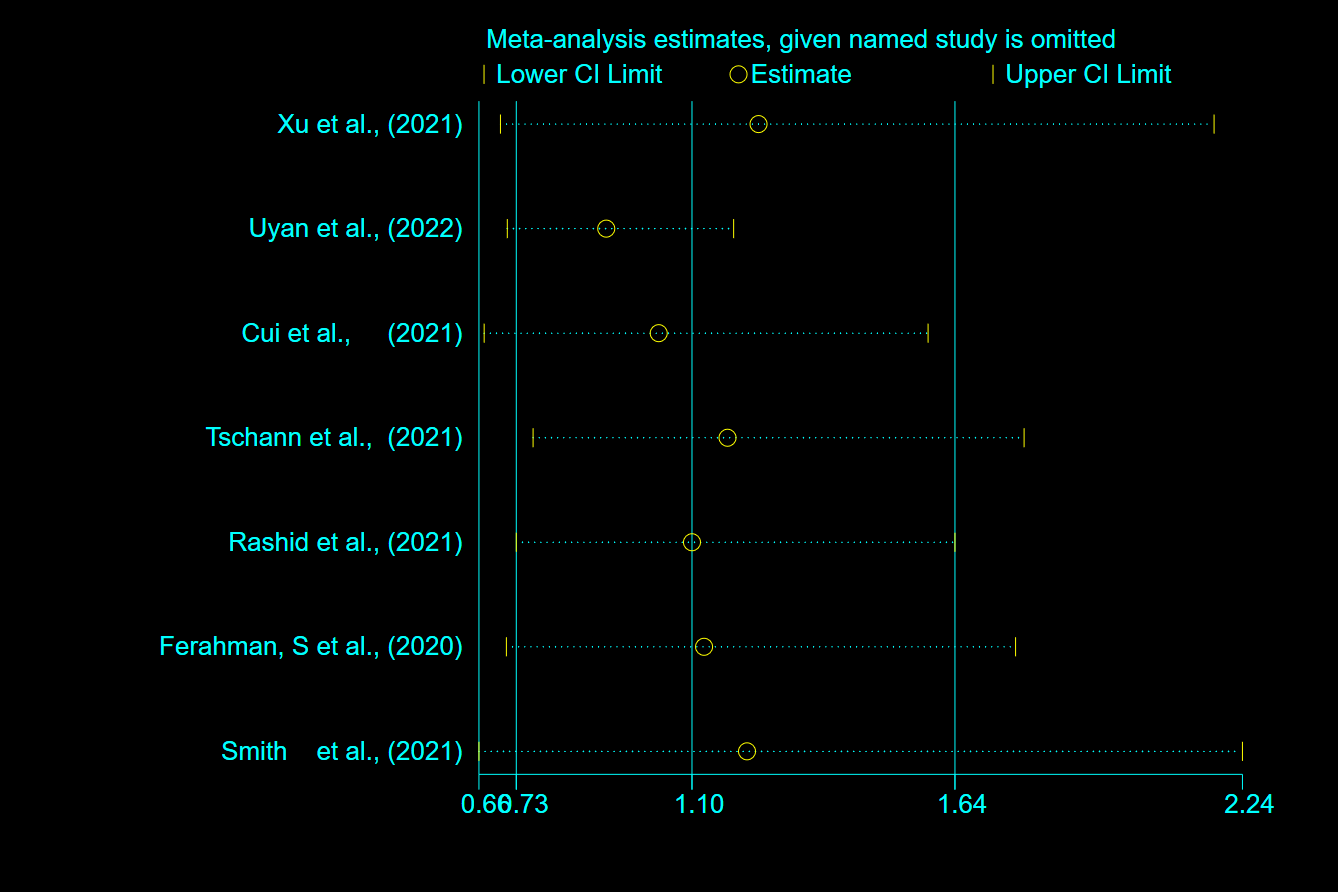

Supplement: Supplementary file 2 [file DataSheet_2.docx]
